# Intestinal arteriovenous malformation managed with palliative ligation and division of the feeding artery: a case report

**DOI:** 10.3389/fped.2025.1496089

**Published:** 2025-07-31

**Authors:** Edgar D. Sy, Wei-Hsun Lu, Yi-Sheng Liu, Yan-Shen Shan

**Affiliations:** ^1^ Section Pediatrics Surgery, National Cheng Kung University Hospital, Tainan, Taiwan, China; ^2^ Department of Surgery, National Cheng Kung University Hospital, Tainan, Taiwan, China; ^3^ Department of Radiology, National Cheng Kung University Hospital, Tainan, Taiwan, China

**Keywords:** arteriovenous malformation (AVM), intestinal AVM, hematochezia, ligation and division of artery, CT arteriography

## Abstract

Arteriovenous malformation (AVM) is a congenital vascular anomaly characterized by an abnormal connection between arteries and veins, bypassing the capillary system. AVM is commonly found in the central nervous system, as well as in the peripheral vascular system and the gastrointestinal tract. Symptomatic intestinal AVM (I-AVM) may present as gastrointestinal bleeding, ranging from occult bleeding to hematochezia. Diagnosis depends on the severity of symptoms, hemodynamic status, and location and size of the lesion and involves either endoscopy, arteriography, or contrast-enhanced computed tomography. Treatment varies based on the diagnostic modality used, such as endoscopic coagulation/clipping, trans-arterial embolization, or intestinal resection. Bleeding I-AVM located in the terminal ileum and presenting with a single prominent feeding artery can be managed with palliative ligation and division of the feeding artery rather than using bowel resection to preserve the ileocecal valve. Lifetime clinical follow-up is necessary due to the recurrence of bleeding secondary to vessel interconnection via the vasa recta and non-degeneration of the AVM nidus.

## Introduction

The etiology of gastrointestinal (GI) bleeding in the pediatric population poses a difficulty in diagnosis. Arteriovenous malformation (AVM) is a congenital vascular disease caused by an abnormal connection between arteries and veins, bypassing the capillary system. In contrast, acquired angiodysplasia of the gastrointestinal tract is caused by a degenerative process resulting from chronic and intermittent contraction of the intestine, leading to obstruction of the venous drainage of the mucosa (1). AVM can occur anywhere in the body. Symptomatic intestinal AVM (I-AVM) typically presents as gastrointestinal bleeding. Clinical signs and symptoms vary depending on the severity of bleeding, ranging from occult to hematochezia, anemia, and hemodynamic instability. Various diagnostic modalities can be used, such as endoscopy (2), arteriography (3), enhanced computed tomography (CT) (4), and laparotomy. Treatment options include endoscopic coagulation/clipping, trans-arterial embolization, ligation and division of the feeding artery (LDFA), and bowel resection. Herein, we present a case of I-AVM successfully managed with LDFA.

## Case presentation

**Figure 1:**
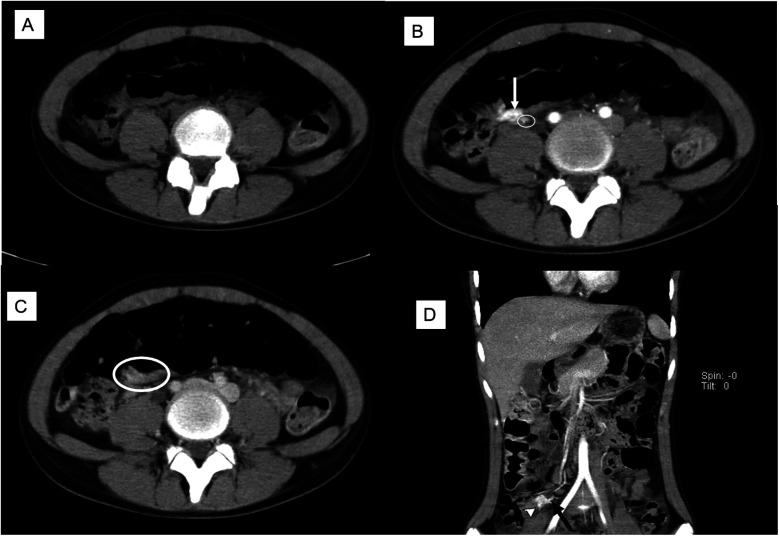
Preoperative CT scan with cross-link. **(A)** Non-contrast phase: no lesion noted. **(B)** The arterial phase showed a 2.5 cm enhanced lesion (arrow) with a suspected feeding artery (circle). **(C)** Delayed phase: a suspect hypo-enhanced lesion (circle). **(D)** Coronal view showed an enhanced lesion (arrowhead) and a feeding artery (arrow).

**Figure 2:**
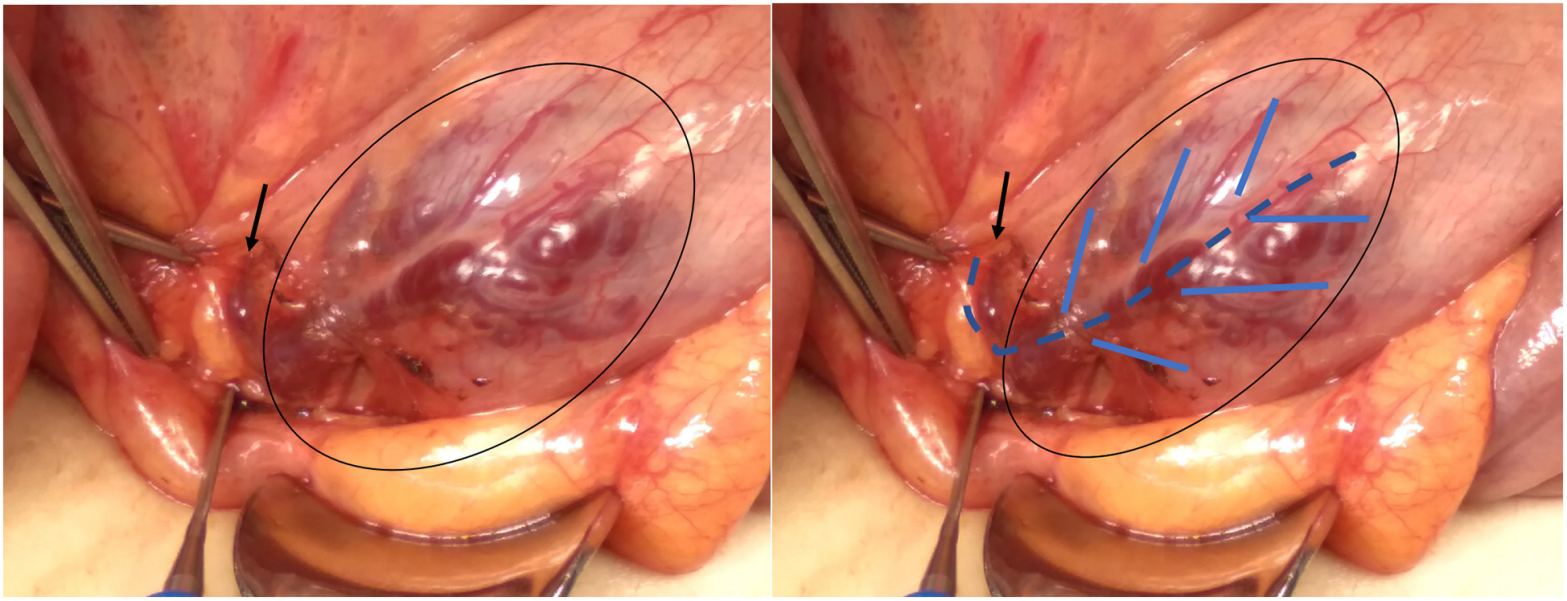
Intraoperative findings showed a subserosal vascular lesion, involving approximately 2.5 cm of terminal ileum just above the IC junction, which resembles a leaf with a petiole located distally. The open mesentery showed a prominent feeding vessel (black arrow).

**Figure 3:**
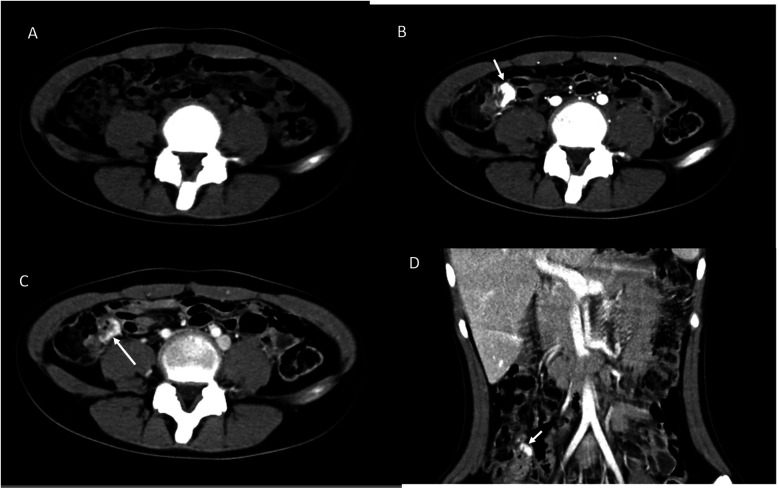
Postoperative CT scan after 6 months with cross-link. **(A)** Non-contrast phase showed no lesion. **(B)** The arterial phase showed enhanced lesion in the coronal view **(D)**. **(C)** The delayed phase showed decreased enhancement of the lesion (arrow).

A 13-year-old girl presented with intermittent passage (5–6 times) of bloody stool (hematochezia) accompanied by low abdominal pain for 3 days. She reported one episode of nausea, vomiting of non-coffee ground residual food substance, poor appetite, and a mild fever. Over the past 10 years, there was no history of bloody stools, abdominal pain, or intake of hormone-based medications. She was brought to a hospital where she had syncope. On physical examination, she appeared pale with dry oral mucosa and hyperactive bowel sounds without abdominal tenderness. Blood examination revealed a hemoglobin (Hb) level of 5.5 gm/dl. Blood transfusion using packed red blood cells (PRBC) was performed. Computed tomography (CT) showed a contrast-enhanced vascular lesion in the distal small bowel with a suspected feeding vessel in the arterial phase (Figure 1). The patient was subsequently transferred to our hospital for further management. Her initial vital signs were as follows: blood temperature (BT), 36.2°C; heart rate (HR), 79 bpm; respiration rate (RR), 20 breaths/min; blood pressure (BP), 113/73 mmHg; and oxygen saturation (SaO_2_), 98%. Repeat blood examination showed a Hb level of 9.8 gm/dl with a hematocrit level of 28.5 vol%. Initial management included close observation, nil per os, proton pump inhibitor, and tranexamic acid infusion. However, on the next day, she had an episode of positional hypotension and passed a large amount of hematochezia. Hb dropped to 6.6 gm/dl. Blood transfusion was given, and a surgical exploratory laparotomy was performed with the aim of intraoperative enteroscopy. Intraoperative findings showed a grossly vascular lesion, approximately 2.5 cm in length, on the surface of the terminal ileum. The lesion resembled a leaf with a petiole located distally. The mesentery at the distal part of AVM was open, and a prominent feeding vessel was noted (Figure 2). The vessel was further dissected free for 1–2 cm, and no gross branches were noted. LDFA was performed, instead of wide bowel resection and intraoperative enteroscopy, to preserve the ileocaecal valve. One of the engorged vessels was open, with resultant collapse of the whole vascular lesion. Following suture closure of the opened vessel, the lesion did not engorge, indicating the relief of venous hypertension. An additional 30 min of observation was done for bowel ischemia. She was discharged in stable condition with the precaution of future recurrence of GI bleeding due to the vessel interconnection via the vasa recta and non-degeneration of the AVM nidus. At 6 months postoperatively, a follow-up CT still showed a well-enhanced and hypo-enhanced lesion in the terminal ileum during arterial and delayed phase, respectively (Figure 3). However, there were no episodes of hematochezia, and the last Hb was 12.5 gm/dl.

## Discussion

Arteriovenous malformation (AVM) is always congenital in nature, in which there is an abnormal connection between arteries and veins, bypassing the capillary system. It is clinically common especially in the central nervous system; however, it can appear anywhere including the gastrointestinal (GI) tract. Vascular lesions within the GI tract may be congenital or acquired as a result of a degenerative process due to chronic intermittent low-grade obstruction of the muscular vascular unit to venules, capillaries, and arteries, resulting in incompetent precapillary sphincters, which leads to the formation of small arteriovenous communication (1).

AVM is characterized radiographically as a tangle of blood vessels with a central nidus, where abnormal arterial–venous communication exists without a normal intervening capillary bed. It can arise anywhere in the body and have a wide range of presentations, ranging from an asymptomatic birthmark to a life-threatening impingement on vital structures and hemorrhage.

AVMs are thought to be quiescent; however, these lesions might expand over time. The presence of various receptor expression, such as FSHR, GHR, ER, PGR, and androgen receptor, explains the sudden growth of lesions during the growth and development of pediatric patients. Hormonal changes such as follicle-stimulating hormone, growth hormone, progesterone, estrogen, and testosterone occur, which affect the growth and expansion of AVM by increasing neovascularization and endothelial proliferation (5–10).

Vascular malformations have traditionally been divided into low flow (capillary, venous, lymphatic, or mixed lesions) or high flow (lesions with an arterial component) according to blood flow characteristics (11, 12). Various classifications of AVM are reported in literature, such as Hamburg system (13, 14), Houdart classification (15), Moore classification (3), Schobinger clinical assessment (16), Cho–Do classification (17), Yano classification (2), Huprich classification (4), and Yakes classification (18), which are based on clinical and diagnostic modalities such as endoscopy, angiography, and computed tomography. The gold standard for establishing the diagnosis of AVM is angiographic examination, which provides information about the extent of the lesion and hemodynamic status. In symptomatic I-AVM, various diagnostic options can be used, such as endoscopy, radiology, laparotomy, or a combination of the above, and the choice usually depends on the severity of symptoms and hemodynamic status.

In hemodynamic stable AVM, the gold standard for diagnosis is to use endoscopic examination. Yano et al. (2) classified the small-intestinal vascular lesions based on the characteristic appearance (punctuate/pulsatile/patchy erythema), size (1 mm or >1 cm), and with or without venous dilatation or bleeding. Different types of endoscopic examination can be used depending of the site lesion, such as push enteroscopy (19), deep enteroscopy (20), single or double balloon small bowel endoscopy (21, 22), colonoscopy (23), capsule endoscopy (24), or intraoperative enteroscopy (25, 26). Failure of endoscopic examination in demonstrating the source of the bleeding vessel should undergo either classic or CT angiography. Classic angiography can effectively identify vascular lesions as the source of bleeding with the advantage of embolization (27), while multiphase CT angiography (28) is considered a less invasive, accurate, and cost-effective tool for the precise location of lesions (29). Using CT enterography, Huprich et al. (4) classified lesions into three categories: angioectasias, arterial lesions, and venous abnormalities. The presence of contrast extravasation is indicative of active bleeding, presenting as either dispersion, pooling, or expansion of intraluminal contrast medium in the subsequent phase.

The inherent risk for bleeding in I-AVM necessitates therapeutic intervention. Symptomatic bleeding may range from occult bleeding, hematemesis, or hematochezia, with or without hemodynamic instability and signs of anemia. Spetzler et al. (30) reported that the mean difference between mean arterial blood pressure and the feeding artery pressure of intracranial AVM that bleed was smaller, compared with non-bleeding (6.5 mmHg, with a range of 2–15 mmHg, vs 40 mmHg, with a range of 17–63 mmHg), suggesting that higher pressure can result in AVM hemorrhage. Lesion arterialization results in venous hypertension, and exposed I-AVM intraluminal can rupture due to high pressure, vascular wall thinning, or erosion ulceration. Generally, management varies at the time of diagnosis, which depends on the mode of examination, site/size of lesions, and hemodynamic stability. These may include closed observation, endoscopic electrocoagulation/clipping, endovascular embolization, surgical bowel resection, or LDFA.

In the literature review, therapeutic endoscopy can control the bleeding in 90% of patients either using coagulation or clipping (22), while in endoscopic inaccessible lesions, angiography is a valuable alternative for diagnosis and treatment (11). Angiographic embolization of the abnormal feeding arteries blocks and decreases blood flow to the AVM. However, the presence of multiple collaterals may require extensive embolization with the inherent risk of ischemia, tissue necrosis, ulceration, recurrent bleeding (27), or risk of coil migration (31).

Currently, surgical intervention is reserved for cases of therapeutic endoscopic or angiographic intervention failure and should be guided by preoperative localization. Various techniques can be used such as endoscopic Indian ink tattooing/clips (32), angiographic metallic coil application (33), or percutaneous CT-guided injection of methylene blue (34) while, intraoperatively, either using ICG, intravenous injection (35), selective angiography (28, 36), or enteroscopy (37). Surgical removal of an AVM cannot be expected to be curative, and the causes of recurrent bleeding after surgery may include leftover AVM, incomplete resection, occult AVMs missed on arteriography, and appearance of new AVMs postoperatively (38).

As in our case, contrast CT showed a suspected vascular lesion with a feeding vessel and intraoperative finding of a vascular complex on the serosal layer of the bowel with a feeding vessel, making LDFA a management option. Following LDFA, the collapse of the engorged AVM lesion is direct evidence of low flow compared with its previous high flow state, indicating relief of venous hypertension and resembling the effect of trans-arterial embolization. The postoperative 6-month CT angiographic study showing a contrast-enhanced lesion is direct evidence of non-degeneration of the AVM nidus and vessel interconnection via the vasa recta. Clinical follow-up for signs of recurrent bleeding in the future is necessary.

## Conclusion

With the aim of preserving the ileocaecal valve, I-AVM located in the terminal ileum, presenting with high flow and a single feeding artery, can be safely managed with palliative LDFA. Lifetime follow-up is essential due to the recurrence of bleeding secondary to the presence of vessel interconnection via the vasa recta and non-degeneration of the AVM nidus.

## Data Availability

The original contributions presented in the study are included in the article/Supplementary Material, further inquiries can be directed to the corresponding author.
